# Association between Epicondylitis and Cardiovascular Risk Factors in Pooled Occupational Cohorts

**DOI:** 10.1186/s12891-017-1593-2

**Published:** 2017-05-30

**Authors:** Kurt T. Hegmann, Matthew S. Thiese, Jay Kapellusch, Andrew Merryweather, Stephen Bao, Barbara Silverstein, Eric M. Wood, Richard Kendall, James Foster, David L. Drury, Arun Garg

**Affiliations:** 1 Rocky Mountain Center for Occupational and Environmental Health (RMCOEH), School of Medicine, University of Utah, 391 Chipeta Way Suite C, Salt Lake City, UT 84108 USA; 2 Department of Occupational Science & Technology, College of Health Sciences, University of Wisconsin-Milwaukee, PO Box 413, Milwaukee, WI 53201 USA; 3 Department of Mechanical Engineering, University of Utah, 1495 East 100 South, Salt Lake City, UT 84112 USA; 4 Safety and Health Assessment and Research for Prevention (SHARP) Program, 243 Israel Road SE Bldg 3, Tumwater, WA 98501 USA; 5 Physical Medicine and Rehabilitation, University of Utah, 30 North 1900 East, Salt Lake City, UT 84132 USA; 6 United Occupational Medicine, 9555 76th St., Pleasant Prairie, WI 53158 USA; 7 Clement J Zablocki VA Medical Center, Compensation & Pension Department, 5000 W National Avenue, Milwaukee, WI 53295 USA

## Abstract

**Background:**

The pathophysiology of lateral epicondylitis (LE) is unclear. Recent evidence suggests some common musculoskeletal disorders may have a basis in cardiovascular disease (CVD) risk factors. Thus, we examined CVD risks as potential LE risks.

**Methods:**

Workers (*n* = 1824) were enrolled in two large prospective studies and underwent structured interviews and physical examinations at baseline. Analysis of pooled baseline data assessed the relationships separately between a modified Framingham Heart Study CVD risk score and three prevalence outcomes of: 1) lateral elbow pain, 2) positive resisted wrist or middle finger extension, and 3) a combination of both symptoms and at least one resisted maneuver. Quantified job exposures, personal and psychosocial confounders were statistically controlled. Odds ratios (ORs) and 95% Confidence Intervals (CIs) were calculated.

**Results:**

There was a strong relationship between CVD risk score and lateral elbow symptoms, resisted wrist or middle finger extension and LE after adjustment for confounders. The adjusted ORs for symptoms were as high as 3.81 (95% CI 2.11, 6.85), for positive examination with adjusted odds ratios as high as 2.85 (95% CI 1.59, 5.12) and for combined symptoms and physical examination 6.20 (95% CI 2.04, 18.82). Relationships trended higher with higher CVD risk scores.

**Conclusions:**

These data suggest a potentially modifiable disease mechanism for LE.

## Background

The prevalence, incidence, pathophysiology and risk factors of epicondylitis are inadequately defined. The reported prevalence rate of lateral epicondylitis (LE) ranges widely from 0.2% to 41.2% [1–18]. A few studies have estimated LE incidence rates based on either infrequent observations or clinic data with reported annual incidences ranging from 0.9–1.7%, but likely underestimating the true incidence rates primarily due to the infrequency of observations [3, 19–21]. One study of workers from 10 employment settings, that include a minority of workers in this report, were followed monthly for up to 6 years reported a baseline point prevalence of 7.3%, lifetime prevalence rate of 17.2% and an incidence rate of 3.67 per 100 person-years [22]. An incidence rate as high as 11.3% has been reported [23]. These wide-ranging estimates may be partially explained by heterogeneity of study methods including differences in intensity of surveillance methods, populations studied, and case definitions.

The pathophysiology of LE is also unclear, with purportedly several competing explanatory pathophysiological findings. These include: hyperlaxity [24], posterolateral rotatory instability [25], myofascial pain [26], trigger points [26, 27] and extensor carpi radialis longus tears and granulation tissue [28–33]. As the pathophysiology is unclear, it may be unsurprising that physical examination findings, treatment options and surgical techniques vary considerably and at times appear contradictory, e.g., beneficial effects of rest vs. exercise, botulinum injections vs. exercise, and ligament cutting vs. aponeurotic release [10, 27, 29, 34–38].

Longitudinal studies suggest increasing age [12, 19, 39–41], and obesity [19] may be LE risk factors. Longitudinal data also suggest low social support [19, 42] and depression [41] are risks, although one study found no increased risk attributable to low social support [4]. Genetic factors are also reportedly risks [43, 44].

There are longstanding reports that LE is associated with forceful athletic use [45–51], although the study methods used have mostly been retrospective. Job physical factors have been largely evaluated by self-report and/or in retrospective studies [1, 2, 9, 12, 16–18, 20, 39, 41, 52–62]. Such methods are prone to produce associations based on “common beliefs” that are not likely to be dispositive regarding determinations of true risk factors. Thus, the mechanistic understanding of LE is fairly primitive.

A systematic review reported that shoulder pain is associated with CVD risk factors [63]. A recent publication also suggested CVD risks are carpal tunnel syndrome (CTS) risks with odds ratios over 5-fold for CTS and over 8-fold for abnormal median nerve conduction [30].

The purpose of this study is to evaluate the potential for association(s) between cardiovascular disease risk factors and LE separately in a large pooled study of three prospective cohort studies involving systematic data collected from over 1800 workers in 35 workplaces in 4 US states.

## Methods

This report is of the baseline, cross sectional data for these prospective cohort studies. Institutional Review Boards approved the study at the University of Utah (11889), the University of Wisconsin-Milwaukee (#03.02.059) and the State of Washington (A-050900-L). Data were collected from 2002 to 2006 and data analysis was conducted in 2015 and 2016.

Worker recruitments were conducted at 35 facilities involving 25 diverse industries located in the states of Illinois, Utah, Washington and Wisconsin beginning in 2001 through 2007. Industries included were manufacturing, food processing, and office jobs. Workers were consented. Workers were recruited regardless of the presence or absence of symptoms until pre-determined enrollment targets based on sample size calculations were met. The only exclusions were marked hand deformities and severe inflammatory arthritides.

Health data were collected by the Health Outcomes Assessment Teams using computerized questionnaires and structured interviews. Standardized physical examinations were conducted. Questionnaire data included age, gender, hobbies, exercise habits, job satisfaction, depression symptoms, diabetes mellitus, and hypertension. Structured interviews utilized symptoms diagrams for anatomically localizing pain. The presence and distribution of pain was captured by location. Body mass indices (BMIs) were calculated from measured heights and weights. Blood pressure was measured using automatic cuffs after being seated for at least five minutes (Omron HEM-780).

Data collected for this study’s health outcomes on all subjects regardless of symptoms were: (i) lateral elbow pain, (ii) resisted wrist extension and/or resisted middle finger extension, and (iii) a case definition for LE that required both lateral elbow pain and at least one of the two positive resisted examination maneuvers.

Job Evaluation Teams measured and videotaped the worker’s job(s). Jobs were measured for six primary factors: force, repetition rate, duration of exertion, posture, speed of work, and task duration per day. Strain Index scores, a composite measure of physical job strain, were computed from those six factors [64–66].

Framingham Heart Study’s heart disease risk model is a sex-specific model that incorporates multiple cardiovascular disease risk factors that have been validated as predictive of 10-year risk of coronary artery disease: age, sex, hypertension, systolic blood pressure, smoking, total cholesterol and HDL cholesterol [67]. For the Framingham model, point values, stratified by gender, were assigned for variables of age, treated and untreated measured or self-reported past diagnosis of hypertension, tobacco use and diabetes mellitus (Table 1). Modified values were used as blood pressure was measured for participants in Illinois, Utah and Wisconsin (Washington did not measure blood pressure, *n* = 749 missing measurements), and cholesterol was excluded from the scoring as it was not measured. Workers without a blood pressure measurement but with a history of hypertension were conservatively assigned a blood pressure value of 1 point. Each worker’s CVD risk score was calculated by summing the individual CVD variable point values. Individualized CVD risk scores range from 0 to 29. An a priori decision was made without knowledge of the relationships to LE to collapse scores ≥16 into one category, as scores above 16 were too infrequent to provide accurate statistical power. Additional analyses of the risk from the Framingham risk model on the Illinois, Utah and Wisconsin data were performed that included blood pressure measurements, hypertensive history and cholesterol history.

**Table 1:** Modified Framingham risk profiles by gender

Score	Age, y	High Cholesterol	Systolic BP + No High BP diagnosis	Systolic BP + Yes High BP diagnosis	Tobacco use	Diabetes
CVD Risk Scores for Women						
0	≤34.9	No	<130	<120	No	No
1			130–139			
2	35–39.9		140–149	120–129		
3		Yes		130–139	Yes	
4	40–40.9		150–159			Yes
5	45–49.9		≥160	140–149		
6				150–159		
7	50–54.9			≥160		
8	55–59.9					
9	60–64.9					
10	65–69.9					
11	70–74.9					
12	≥75					
CVD Risk Scores for Men						
0	≤34.9	No	<130	<120	No	No
1			130–139			
2	35–39.9	Yes	140–159	120–129		
3			≥160	130–139		Yes
4				140–159	Yes	
5	40–40.9			≥160		
6	45–49.9					
7						
8	50–54.9					
9						
10	55–59.9					
11	60–64.9					
12	65–69.9					
13						
14	70–74.9					
15	≥75					

*CVD* cardiovascular disease, *BP* blood pressure, *mmHg* Points allotted based on the Framingham Heart Study CVD risk tables

### Statistical analyses

The risk between individualized CVD risk score was analyzed separately for the three health outcomes prevalences of (i) lateral elbow pain, (ii) at least one resisted examination maneuver and (iii) LE using logistic regression. These are hereafter referred to as pain, examination findings and LE, respectfully. Missing data were minimized by using computerized instruments. Univariate analyses were done with each variable individually to conclude separate associations with each of the three health outcomes and then combined in a multivariate logistic regression to assess the influence of confounders for each health outcome. Statistical significance is *p* < 0.05. Variables with meaningful evidence of associations with LE (*p* < 0.20) were considered for inclusion in multivariate models as potential confounders. These potential confounders included job physical exposures (Strain Index for the typical job task on the right hand), BMI, and job satisfaction. Assessments were made for collinearity between potential confounders. The final main effects model included all confounders that were statistically significant or had an epidemiological basis for a causal relationship and were trending toward statistical significance (*p* < 0.20).

## Results

The population consisted of 1824 workers, of which 1088 (59.6%) were female (see Table 2). The mean age was 41.1 ± 11.4 years. Minorities of workers had diabetes mellitus (*n* = 86, 4.7%), hypertension (*n* = 288, 15.8%), and had ever smoked (729, 40.0%). The mean body mass index was 28.7 ± 6.5 kg/m^2^.

**Table 2:** Descriptive and demographic data of the pooled studies at baseline (*n* = 1824) N(%)

Variable^a^	Symptoms at time of exam^b^	Positive exam findings^c^	Lateral epicondylitis (symptoms and exam)^d^	No lateral epicondylitis	Total
(*N*=)	(*n* = 273, 15.0%)	(*n* = 264, 14.5%)	(*n* = 121, 6.6%)	*n* = 1703, 93.4%)	(*n* = 1824, 100%)
Age (years)	43.4 (9.6)	43.6 (9.8)	44.5 (8.6)	40.9 (11.5) *p* = 0.001	41.1 (11.4)
Gender				*p* = 0.008	
Female	199 (72.9%)	183 (69.3%)	86 (71.1%)	1002 (58.8%)	1088 (59.6%)
Male	74 (27.1%)	81 (30.7%)	35 (28.9%)	701 (41.2%)	736 (40.4%)
Diabetes Mellitus				*p* = 0.144	
Yes	18 (6.6%)	15 (5.7%)	9 (7.4%)	77 (4.5%)	86 (4.7%)
No	255 (93.4%)	249 (94.3%)	112 (92.6%)	1626 (95.5%)	1738 (95.3%)
Hypertension				*p* = 0.207	
Yes	43 (15.8%)	50 (18.9%)	24 (19.8%)	264 (15.5%)	288 (15.8%)
No	230 (84.2%)	214 (81.1%)	97 (80.2%)	1439 (84.5%)	1536 (84.2%)
Average systolic Blood Pressure (mmHg)	128.9 (17.9)	128.2 (17.8)	130.0 (18.7)	12.5 (17.1) *p* = 0.518	127.7 (17.2)
Tobacco Use				*p* = 0.097	
Never	152 (55.7%)	155 (58.7%)	64 (52.9%)	1031 (60.5%)	1095 (60.0%)
Ever	121 (44.3%)	109 (41.3%)	57 (47.1%)	672 (39.5%)	729 (40.0%)
Body Mass Index (kg/m2)	29.8 (6.7)	28.5 (6.7)	29.4 (6.7)	28.6 (6.5) *p* = 0.157	28.7 (6.5)
CVD Risk Score	6.8 (3.9)	6.6 (3.9)	7.3 (4.0)	5.8 (4.2) *p* = 0.0003	5.9 (4.2)

^a^N(%) for categorical variables. Mean (Standard Deviation) for continuous variables

^b^Lateral elbow pain

^c^Either lateral elbow pain with resisted wrist extension or middle finger extension

^d^Case definition of lateral epicondylitis with both Lateral elbow pain and at least one resisted wrist physical examination maneuver

A total of 273 (15.0%) had lateral elbow symptoms at baseline. A positive examination finding of either resisted wrist extension or resisted middle finger extension was present in 264 (14.5%). Lateral epicondylitis, defined by both lateral elbow symptoms and a resisted examination maneuver, was present in 121 (6.6%), which is this population’s point prevalence rate.

The mean age was greater among those with symptoms, examination findings or having LE (OR per year = 1.02, *p* < 0.0004, 1.02 *p* < 0.0001, and 1.03, *p* < 0.001 respectively). The population had more females than males (*n* = 1088, 59.6%), and modestly higher risk of LE with female sex OR = 1.72, 95% CI 1.15, 2.58 (*p* = 0.008). Diabetes mellitus was present in 86 (4.7%), but was present in 7.4% in those with LE (OR = 1.70, 95% CI 0.83, 3.47, *p* = 0.148). Hypertension was present in 288 (15.8%) and modestly more common among those with LE (OR = 1.35, 95% CI 0.85, 2.15). Among the 1075 participants who had measured blood pressure, the systolic blood pressure was somewhat higher in the LE case group 130.0 ± 18.7 mmHg compared with the non-LE case group at 127.5 ± 17.1 mmHg. The Body Mass Index (BMI) was higher in the LE group 29.4 ± 6.7 compared with the non-LE case group’s BMI of 28.6 ± 6.5 kg/m^2^. The overall mean individualized CVD risk score was higher in the LE case group 7.3 ± 4.0 compared with the non-LE case group 5.8 ± 4.2 (*p* = 0.0003).

Data were analyzed to assess associations between the person’s CVD risk factor score and risk of lateral elbow pain, examination findings and LE (see Table 3). Separate analyses assessed relationships between CVD risk factor scores and 1) lateral elbow pain regardless of test findings, 2) at least one positive physical examination test result (either resisted middle finger or wrist extension) and 3) LE as defined by both symptoms and at least one positive physical examination test result. For unadjusted associations, there was a trend of increasing risk for both LE symptoms and positive test result with CVD risk factor scores peaking at odds ratios of 3.61 (95% CI 2.02, 6.47) and 2.81 (95% CI 1.57, 5.01), respectively. For analyses of risk for LE, the results showed mostly stronger associations than for symptoms or examination test alone and peaked at an odds ratio of 6.62 (95% CI 2.21, 19.80). Body mass indices were significantly related to LE symptoms. The Strain Index scores that assessed job physical demands were significantly related to positive physical examination test results in the univariate analyses. Job dissatisfaction had significant univariate association with both LE symptoms and LE, but not with positive physical examination test results.

**Table 3:** Crude OR (95% CI) for right lateral elbow symptoms, right lateral elbow physical examination maneuvers and right lateral epicondylitis*

Crude Analyses	Lateral Elbow Symptoms	Positive Resisted Elbow or Middle Finger Extension	Lateral Epicondylitis (Symptoms plus at least one Exam Maneuver)
Framingham score	OR (95% CI)	OR (95% CI)	OR (95% CI)
0	1.00 (Reference)		1.00 (Reference)
1	0.77 (0.17, 3.43)	0.34 (0.04, 2.58)	N/A
2	**2.13 (1.09, 4.15)**	1.80 (0.93, 3.51)	**3.62 (1.26, 10.4)**
3	2.05 (0.98, 4.28)	1.22 (0.54, 2.75)	2.65 (0.79, 8.88)
4	1.58 (0.85, 2.92)	**2.21 (1.26, 3.88)**	2.03 (0.71, 5.79)
5	**2.47 (1.37, 4.46)**	**2.33 (1.31, 4.13)**	**4.60 (1.78, 11.9)**
6	**2.68 (1.35, 5.33)**	1.77 (0.86, 3.64)	**4.18 (1.41, 12.4)**
7	**3.43 (1.89, 6.23)**	**2.04 (1.09, 3.81)**	**3.45 (1.23, 9.69)**
8	**3.61 (2.02, 6.47)**	**2.81 (1.57, 5.01)**	**4.91 (1.86, 12.9)**
9	**2.46 (1.22, 4.93)**	**2.23 (1.12, 4.42)**	**4.14 (1.40, 12.2)**
10–12	**1.99 (1.12, 3.53)**	**1.99 (1.15, 3.45)**	**3.19 (1.23, 8.29)**
13–15	**2.62 (1.35, 5.10)**	**2.08 (1.06, 4.07)**	**4.64 (1.64, 13.1)**
16+	**2.89 (1.34, 6.24)**	**2.36 (1.08, 5.13)**	**6.62 (2.21, 19.8)**
Per Unit for Framingham	**1.05 (1.02, 1.08)**	**1.05 (1.02, 1.08)**	**1.08 (1.03, 1.12)**
Body Mass Index	**1.03 (1.01, 1.05)**	0.99 (0.97, 1.02)	1.02 (0.99, 1.05)
Strain Index	1.00 (0.99, 1.01)	**0.98 (0.96, 0.99)**	0.98 (0.95, 1.00)
**Job satisfaction**			
Satisfied	1.00 (Reference)	1.00 (Reference)	1.00 (Reference)
Neither satisfied or Dissatisfied	**1.62 (1.16, 2.25)**	1.22 (0.89, 1.67)	**1.80 (1.10, 2.94)**
Dissatisfied	**2.06 (1.40, 3.02)**	1.26 (0.86, 1.84)	**2.01 (1.14, 3.54)**

*OR* Odds ratios, *C* Confidence interval, *RLES* right lateral elbow symptoms, *RLE* Right lateral epicondylitis

*The physical exam consisted of either a resisted wrist or middle finger extension. RLE was based on combined symptoms and at least one resisted maneuver

**Fig. 1:**
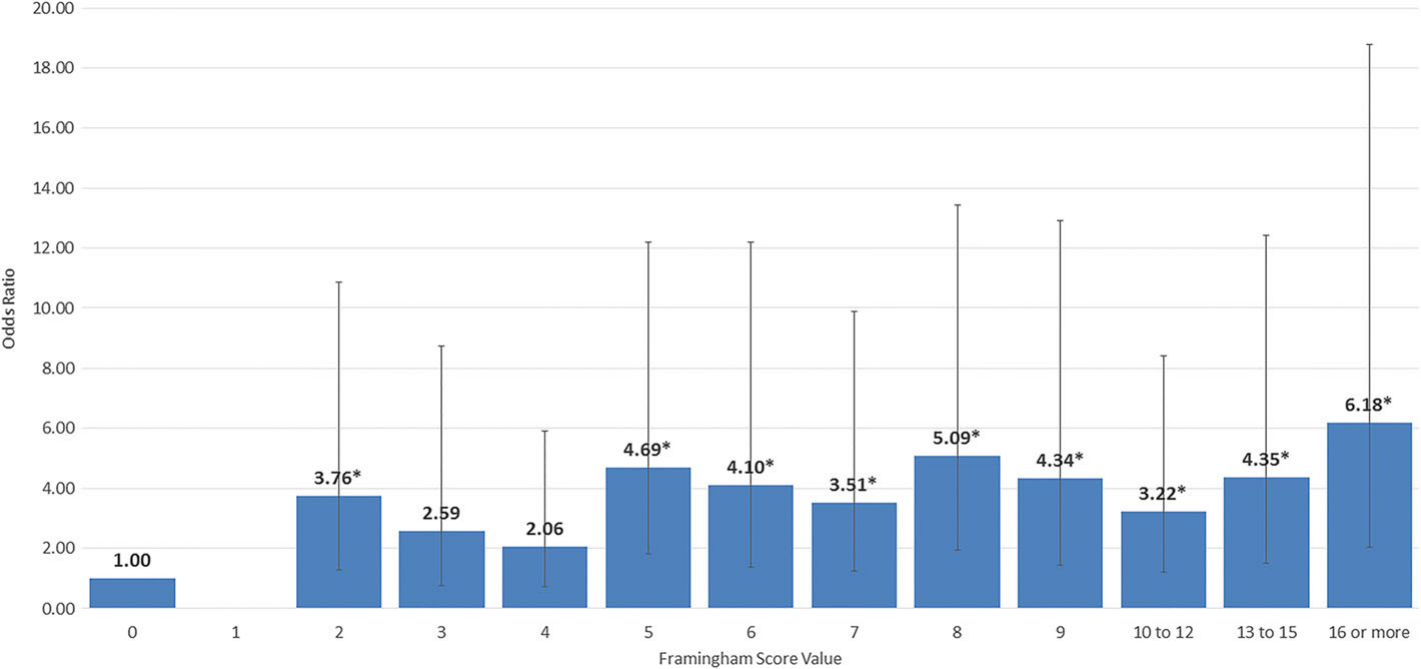
Odds ratios and 95% confidence intervals for lateral epicondylitis by cardiovascular disease risk score adjusted for body mass index, strain index and job satisfaction

Adjusted analyses were performed that included BMIs, Strain Index scores and job satisfaction (see Table 4). These results were largely comparable to the unadjusted rates. Risk of LE rose across the cardiovascular disease risk scores in a highly significant trend (*p* = 0.0005) (see Fig. 1). The peak risk of LE was an OR of 6.20 (95% CI 2.04, 18.8). The point estimates were lower for either symptoms alone or physical examination findings alone. BMIs were not significantly associated with LE. Strain Index was borderline associated. Job satisfaction remained significant with those dissatisfied having 2.34-fold risk of having LE (95% CI 1.31, 4.17).

**Table 4:** Adjusted* OR (95% CI) for right lateral elbow symptoms, right lateral elbow physical examination maneuvers and right lateral epicondylitis**

Adjusted analyses	Lateral Elbow Symptoms	Positive Resisted Elbow or Middle Finger Extension	Lateral Epicondylitis (Symptoms plus at least one Exam Maneuver)
Framingham score	OR (95% CI)	OR (95% CI)	OR (95% CI)
0	1.00 (Reference)	1.00 (Reference)	1.00 (Reference)
1	0.69 (0.15, 3.12)	0.35 (0.05, 2.66)	N/A
2	**2.18 (1.11, 4.28)**	1.86 (0.95, 3.63)	**3.76 (1.30, 10.9)**
3	1.87 (0.89, 3.92)	1.26 (0.56, 2.84)	2.59 (0.77, 8.73)
4	1.57 (0.85, 2.92)	**2.27 (1.29, 3.98)**	2.06 (0.72, 5.91)
5	**2.54 (1.40, 4.61)**	**2.36 (1.33, 4.20)**	**4.69 (1.80, 12.2)**
6	**2.58 (1.29, 5.16)**	1.78 (0.86, 3.69)	**4.10 (1.38, 12.2)**
7	**3.45 (1.89, 6.29)**	**2.10 (1.12, 3.92)**	**3.51 (1.24, 9.89)**
8	**3.81 (2.11, 6.85)**	**2.85 (1.59, 5.12)**	**5.08 (1.92, 13.4)**
9	**2.53 (1.25, 5.11)**	**2.32 (1.16, 4.64)**	**4.34 (1.45, 12.9)**
10–12	**1.95 (1.10, 3.49)**	**2.05 (1.18, 3.57)**	**3.22 (1.24, 8.41)**
13–15	**2.33 (1.18, 4.58)**	**2.13 (1.08, 4.21)**	**4.35 (1.52, 12.4)**
16+	**2.57 (1.18, 5.61)**	**2.42 (1.10, 5.31)**	**6.20 (2.04, 18.8)**
	**1.05 (1.02, 1.09)**	**1.05 (1.02, 1.08)**	**1.08 (1.03, 1.13)**
Body Mass Index	**1.03 (1.01, 1.05)**	0.99 (0.97, 1.01)	1.01 (0.98, 1.04)
Strain Index	1.00 (0.99, 1.01)	**0.98 (0.96, 0.99)**	**0.97 (0.95, 1.00)**
**Job satisfaction**			
Satisfied	1.00 (Reference)	1.00 (Reference)	1.00 (Reference)
Neither satisfied or Dissatisfied	**1.64 (1.17, 2.29)**	1.26 (0.92, 1.74)	**1.86 (1.13, 3.06)**
Dissatisfied	**2.23 (1.51, 3.30)**	1.42 (0.96, 2.10)	**2.34 (1.31, 4.17)**

*OR* Odds ratios, *C* Confidence interval, *RLES* right lateral elbow symptoms, *RLE* Right lateral epicondylitiss

*Adjusted for variables in the table, i.e., Framingham score, body mass index, Strain Index (measure of job physical demand). **The physical exam consisted of either a resisted wrist or middle finger extension. RLE was based on combined symptoms and at least one resisted maneuver

Adjusted analyses were also performed on the subset of Illinois, Utah and Wisconsin data that included blood pressure measurements, hypertensive histories and cholesterol histories. (See Table 5.) Those analyses revealed elevated risks for lateral elbow symptoms with a peak OR of 5.71 (95% CI 2.32, 14.07) and a peak OR for a positive physical examination maneuver of 4.09 (95% CI 1.31, 12.79). The peak risk for lateral epicondylitis was an OR of 8.60 (95% CI 2.17, 34.02).

**Table 5:** Risk of lateral elbow symptoms and lateral epicondylitis associated with Framingham risk scores

Framingham score	OR (95% CI) for current symptoms	OR (95% CI) for physical exam maneuver	OR (95% CI) for case definition of lateral epicondylitis
0	1.00 (Reference	1.00 (Reference	
1	1.54 (0.30, 8.03)	<0.001 (<0.001, >999.999)	
2	3.05 (1.03, 9.01)	1.88 (0.48, 7.39)	1.00* (Reference*)
3	3.09 (1.13, 8.47)	1.78 (0.49, 6.40)	2.89 (0.57, 14.72)
4	2.31 (0.88, 6.06)	2.87 (0.97, 8.50)	3.13 (0.73, 13.40)
5	2.95 (1.16, 7.50)	3.53 (1.24, 10.07)	5.36 (1.41, 20.38)
6	2.03 (0.60, 6.86)	1.67 (0.38, 7.37)	1.40 (0.14, 13.88)
7	3.63 (1.42, 9.24)	1.97 (0.62, 6.27)	2.75 (0.60, 12.60)
8	5.71 (2.32, 14.07)	3.37 (1.15, 9.87)	5.85 (1.51, 22.71)
9	3.67 (1.30, 10.36)	2.14 (0.59, 7.79)	4.91 (1.06, 22.80)
10	3.00 (1.25, 7.18)	2.94 (1.07, 8.07)	3.95 (1.08, 14.48)
13	3.18 (1.22, 8.31)	2.95 (0.97, 9.00)	4.17 (1.01, 17.30)
16	3.83 (1.40, 10.47)	4.09 (1.31, 12.79)	8.60 (2.17, 34.02)

*There were no cases of LE in Framingham Scores of 0 and 1, so we collapsed 0,1,2 into one reference category in order to generate stable estimates

## Discussion

This large, multi-plant, multi-state study found significantly elevated risks of lateral epicondylitis (LE) associated with cardiovascular disease risk factors after adjusting for job physical factors, BMI, and job satisfaction. The magnitude of the association is as high as 6-fold with a strong trend across the CVD risk scores (*p* = 0.0005). This evidence adds to a growing body of evidence that common, soft-tissue musculoskeletal disorders, including shoulder disorders, Achilles tendinopathy, and carpal tunnel syndrome [30, 63, 68, 69], may have pathophysiological bases in CVD risks. A discrete mechanism of action of CVD risk is clearer in the shoulder and Achilles where tenuous blood supply to the tendons is well defined [70–74]. For CTS and lateral epicondylitis, it may be that the CVD risk is similarly associated with reduced blood supply, which increases susceptibility to biomechanical and other factors.

This study found that the individual risk factors that compose the Framingham model (e.g., tobacco, diabetes mellitus, and hypertension) were mostly trending towards significance. That the overall CVD risk factor modeling results that included those same Framingham individual risk factors are so strong suggests the CVD risk factors interact, as is well reported in cardiovascular disease, and thus do meaningfully influence the development of LE [75, 76].

Additionally, that the weaker associations with CVD risks for the lateral elbow symptoms and physical examination findings compared to LE are expected and consistent. A principle of epidemiological research is that the more precise a diagnosis, the stronger is the ability to find effects [77, 78]. The findings for the two other health outcomes (lateral elbow symptoms and physical examination findings) thus support the overall impact of the results for LE.

Individual cardiovascular disease risk factors have been previously reported as risks for LE. A prior case-control study found peripheral vascular disease and diabetes mellitus were both associated with LE [68]. Diabetes mellitus has been reported to be a LE risk [12], as well as chronic hyperglycemia [79]. Three studies have suggested prior smoking was a risk for LE [12, 80, 81]. Two studies reported a trend of increasing LE risk with obesity [12, 19], although another found it to not be a risk [80]. Our study failed to find BMI as a risk factor. That difference may possibly be due to the greater ability to control for more factors in this study. It is noteworthy that the Framingham CVD risk factor model does not include obesity.

This study systematically evaluated the prevalence of lateral elbow symptoms, examination findings and LE. The point prevalence estimate for each of these was 15.0%, 14.5% and 6.6%, respectively. These prevalence estimates are higher than most other reviewed studies and unsurprising considering the carefully structured, individualized interviews and physical examinations. Additionally, a significant proportion of worker’s jobs was physically demanding and may have produced some increases in the prevalence rates.

This line of research may have implications for both clinical care and population management. Should CVD risks be confirmed as significant risks for common musculoskeletal disorders (MSDs), patients presenting with one disorder would likely be candidates for more intensive CVD risk management, potentially to prevent both MSDs and CVD. Additionally, effective health promotion disease prevention programs could have greater efficacy beyond traditional cardiovascular diseases to include MSDs. Still, there is considerable research required before interventional programs could be enacted for purposes of addressing MSD risks as CVD risks.

### Strengths of this study

Strengths of this study include the large sample size, multi-state population, systematic measurement of lateral elbow pain and physical examination findings, measured BMI, and measured blood pressure. The systematic measurement of all these factors in a large population-based study is a unique strength. The systematic approach to use a modified Framingham CVD risk score to quantify cardiovascular risks is another strength. The adjustment for laboriously quantified job physical risk factors is an extraordinarily rare strength and helps to remove that potential confounder.

### Limitations of this study

Weaknesses include the cross sectional design, although the extreme costs to measure, videotape and quantify job physical factors makes a prospective cohort study to duplicate these results with sufficient powering difficult. A cross sectional design largely precludes causal inference. The large proportion of workers from the manufacturing sector is a potential limitation, although this study included workers from the services and healthcare sectors, and it seems unlikely that the source of patients should materially influence the CVD scores or alter their relationships. The primary exposure in this study is a modified Framingham CVD risk score. To address that weakness, we performed the analyses on the subset of data with complete blood pressure measurements, hypertensive histories and cholesterol histories; those analyses also showed strong, meaningful associations between CVD risk and lateral epicondylitis.

Prospective cohort analyses are needed to confirm these results in incidence data and duplicated elsewhere. Studies reporting changes in LE prevalence and/or incidence rates based on CVD risk factor modification are also needed.

## Conclusions

This study suggests there is a strong association between CVD risk score and LE that demonstrates strength of association, consistency with other studies evaluating individual CVD factors, a biological gradient response, and biological plausibility. This association remains after adjustment for known and suspected confounders, including meticulous quantification of job physical factors. These results suggest a strong, potentially modifiable disease mechanism. However, whether CVD risk factor modification reduces risk of LE requires further investigation.

## Abbreviations

BMI: Body Mass Index
CI: Confidence Interval
CTS: Carpal Tunnel Syndrome
CVD: Cardiovascular Disease
LE: Lateral Epicondylitis
MSD: Musculoskeletal Disorder
NIOSH: National Institute for Occupational Safety and Health
OR: Odds Ratio
SI: Strain Index
